# Cognitive dysfunction and cortical structural abnormalities in first-episode drug-naïve schizophrenia patients with auditory verbal hallucination

**DOI:** 10.3389/fpsyt.2022.998807

**Published:** 2022-09-16

**Authors:** Xuran Shen, Fuli Jiang, Xinyu Fang, Wei Yan, Shiping Xie, Rongrong Zhang

**Affiliations:** Department of Psychiatry, The Affiliated Brain Hospital of Nanjing Medical University, Nanjing, China

**Keywords:** schizophrenia, auditory verbal hallucination, cognitive function, cortical thickness, local gyrification index

## Abstract

**Objective:**

The current study aimed to examine the cognitive profiles and cortical structural alterations in first-episode drug-naïve schizophrenia with AVH (auditory verbal hallucination).

**Methods:**

Cortical structural parameters including cortical thickness and local gyrification index (LGI) estimated using FreeSurfer as well as cognitive performance assessed on the MATRICS Consensus Cognitive Battery (MCCB) were acquired from 78 schizophrenia patients with AVH, 74 schizophrenia patients without AVH (non-AVH), and 76 healthy controls (HC). Hoffman Auditory Hallucination Rating Scale (HAHRS) was applied to assess the severity of AVH.

**Results:**

The results revealed extensive deficits in all cognitive domains among AVH, non-AVH, and HC groups. Compared to non-AVH group, the AVH group showed poorer performance on visual learning and verbal learning domains. There were six brain regions with cortical thinning in the right hemisphere of inferior temporal gyrus, superior temporal gyrus, lateral orbito frontal cortex, rostral anterior cingulate cortex, supramarginal gyrus and insula, and two brain regions with increased LGI in the left hemisphere of superior parietal gyrus and the right hemisphere of caudal anterior cingulate cortex on AVH group relative to non-AVH group. Correlation analysis revealed that the cortical thickness in the right hemisphere of lateral orbito frontal cortex was negatively correlated with the severity of AVH in schizophrenia patients with AVH.

**Conclusion:**

Visual learning, verbal learning dysfunction, and specific disruption of cortical structure may characterize schizophrenia patients with AVH during early stages of the disorder. Right lateral orbito frontal cortical deficits may be the pathological mechanisms underlying AVH in first-episode drug-naïve schizophrenia.

## Introduction

Schizophrenia is a persistent deteriorative mental disease with the ambiguity of exact pathogenesis and the high disability rate, which consisting of three core symptoms including positive symptom, negative symptom, and cognitive impairment. Auditory verbal hallucination (AVH) is one of the most cardinal and devastating positive symptom of schizophrenia, with the AVH observed in around 70–80% patients (1, 2). These false auditory perceptions are generally negative or malicious that can lead to aggressive or suicide behaviors in schizophrenia patients, which may threaten the stability of society (3). AVH is also one of the important criteria when diagnosing and judging the severity or clinical efficacy of schizophrenia, and it has been similarly found to be a vital symptom of disease relapses in longitudinal studies (4).

Pharmacological treatments are currently considered as the major treatment of schizophrenia patients suffering from AVH. However, nearly 30% of schizophrenia patients still remain AVH with taking the anti-psychotic medications and are resistant to treatment (5). Despite several decades of explorations, there is currently only weak evidence for the neurobiological mechanisms of AVH in schizophrenia. Previous studies suggested that AVH has something to do with the impairment of cognitive function (6, 7). As the perceptual-inhibitory failure model of AVH, refers to the integration of cognitive explanations in the conceptualization of the AVH phenomenon in schizophrenia, is proposed and gradually recognized (8), the association between AVH and cognitive function has been further verified. Evidence has shown that AVH would aggravate the cognitive impairment of schizophrenia patients, which is more prominent in attention, executive function, working memory and others (6, 7, 9). It was worth noting that cognitive function is consistently recognized as the significant predictor of functional outcomes in the patients with schizophrenia (10). Therefore, the mechanisms by which the AVH exacerbates cognitive impairment and further influences the functional prognosis remain largely unknown.

Evidence from one study suggested that perceptual noise generated spontaneously by the temporal cortex is misidentified as the realistic sound due to the impairment of executive control processes, resulting in auditory hallucinations (6). Pathological change occurred the cortex regions responsible for speech processing and auditory perception, causing the patients hard to accurately recognize whether the auditory signals arise from external or endogenous activities (11, 12). Previous neuroimaging studies implicated structural cortex alterations in the generation of AVH, involving the cortical thickness, cortical surface area or cortical volume (13–16). Lynn et al. focused on the auditory cortex established a significant region of interest (ROI) and reported that schizophrenia patients with AVH had thinner cortex in the left Heschl’s gyrus than patients without AVH (14). A multi-center study has also demonstrated that the thinner cortical thickness in the left middle temporal gyrus was found in a group of schizophrenia with AVH and inversely correlated with AVH severity (15). The consistent finding showed the deficits of basic auditory processing in schizophrenia may correspond with temporal cortex pathology (17). On the basis of the lateralization dysfunction theory of schizophrenia, the generation of AVH may be strongly associated with the dysfunction of the left cerebral hemisphere, especially the left temporal lobe (18). Nonetheless, a limited number of researches have also suggested that reduced cortical thickness in the right Heschl’s gyrus among schizophrenia patients with AVH compared to patients without AVH (19), but still have failed to discover relationships between severity of AVH and cortical thickness in the right cerebral hemisphere.

In addition to cortical thickness pathology, in accordance with the neurodevelopmental hypothesis of schizophrenia, abnormal cortical folding patterns have attracted much attention (20–22). However, cortical thickness in schizophrenia is affected by various pathophysiological processes, which is different from the evolutionary processes affecting cortical folding (23). Local gyrification index (LGI), as a metric measuring cortical folding, was applied to investigate the mechanism of AVH in the current studies, that was refers to the ratio of the cortical surface area buried within the sulcal folds to the external visible cortex (24, 25). Recent studies indicated either increased or reduced cortical folding of schizophrenia in several specific brain regions, such as hyper-gyral patterns in the bilateral anterior cingulate gyrus or prefrontal cortices (21), and hypo-gyral patterns in the precentral gyrus or bilateral posterior cingulate and caudal anterior cingulate (26). Yet, datum on the specificity of abnormal LGI for the patients with AVH are extremely scarce, the mechanisms on cortical folding changes relating to AVH are unclear.

Previous magnetic resonance imaging (MRI) studies were reviewed, the results have explicitly suggested certain brain areas in relation to cognitive impairment (27). For instance, several studies exhibited that the decreased cortical thickness in the frontal and temporal lobes may be correlated with cognitive function abnormalities, mainly performed on attention (28). Another study showed that the loss of cortical thickness in the right lateral orbitofrontal may be related to attention and visual learning (29). In brief, these findings indicated that brain structural correlates of cognitive dysfunction in schizophrenia map onto similar abnormal brain regions implicated in AVH. Therefore, whether AVH and cognitive dysfunction have the consistent brain imaging basis needs to be further verified.

At present, a large number of studies were conducted to explore the neural mechanism of schizophrenia patients characterized by AVH, but previous studies mostly had small sample sizes or chose participants experienced taking medicine (13, 30, 31), which could exert an influence on the brain function and structure. Thus we recruited first-episode and drug-naïve schizophrenia patients to investigate the brain mechanisms underlying AVH. Based on the discrepancies in cognitive characteristics and neurobiological indicators between schizophrenia patients with and without auditory verbal hallucination, we conducted a hypothesis-free analysis to explore the brain imaging mechanisms of AVH exacerbated cognitive impairment in schizophrenia patients.

## Materials and methods

### Participants

A total of 158 patients with first-episode drug-naïve schizophrenia were recruited consecutively from the inpatient department or outpatient clinic in the Affiliated Brain Hospital of Nanjing Medical University. All patients were diagnosed consistently by two experienced senior psychiatrists according to the Diagnostic and Statistical Manual of Mental Disorders (DSM)-5 criteria. After at least 6 months of follow-up, all subjects enrolled in the study were eventually diagnosed with schizophrenia. Inclusion criteria for all schizophrenia patients were as follows: (1) Han ethnicity, right-handed, age between 16 and 45; (2) education years ≥ 8 years, intelligence quotient (IQ) ≥ 70; (3) first episode illness, duration of their first experience of psychosis ≤ 24 months, no taking antipsychotic medications and no physical therapies; (4) the score of 60 or more on the positive scale of the Positive and Negative Syndrome Scale (PANSS). Exclusion criteria included: psychosis associated with alcohol or drug abuse, pervasive developmental disorder and so on, major physical diseases or traumatic brain injury, current pregnant, or breastfeeding, contraindicated and uncooperative patients with magnetic resonance imaging (MRI) scan. Through strict quality control procedures, 152 schizophrenia patients were included in the final analysis.

Seventy-six healthy controls (HC) were recruited from the local area *via* poster advertisements and matched with patients by age and gender. The HC were screened using the Structured Clinical Interview for DSM-IV-TR Axis I, non-Patient Edition (SCID-I/NP), and met the following conditions: Han ethnicity, right-handed, age between 16 and 45; no personal history of psychosis, or a family history of mental disorder. The exclusion criteria were the same as the patients group. In the end, a total of 76 HC completed the assessments and MRI scans.

The study was approved by the Medical Research Ethics Committee of the Affiliated Brain Hospital of Nanjing Medical University. All participants provided written informed consent.

### Clinical assessments

The age, gender, years of education and handedness were obtained from the patients and their parent or guardian as the demographic information. Duration of illness was recorded from age of onset to age at first visit to the doctors. The PANSS was used for Psychopathological assessment in schizophrenia patients, which involved positive symptom, negative symptom and general psychiatric symptom (32). We also obtained available information on whether patients had hallucinations from clinical interviews in PANSS item P3, and assessed AVH severity in all patients with Hoffman Auditory Hallucination Rating Scale (HAHRS) (3). Seventy-eight patients were assigned to AVH group including patients who were experiencing AVH and who had experienced AVH during the course of disease, using HAHRS scores > 0 and P3 scores ≥ 4. Non-AVH group was defined as the patients without AVH during the first episode, and still the absence of AVH at 6 months of follow-up.

### Intelligence quotient and cognitive function

The Chinese version of the Wechsler Adult Intelligence Scale-Revised (WAIS) was applied for measuring the IQ, which included four sub-tests: the common sense, similarity, and picture completion tests, and block design. The MATRICS consensus cognitive battery (MCCB) was used to evaluate the cognitive function in all participants (33), which consisted of seven domains and the total score: speed of processing, attention/vigilance, working memory, visual learning, verbal learning, reasoning/problem solving, and social cognition. A total of nine tests (Trail Making Test, Symbol Coding, Hopkins Verbal Learning-Revised, Spatial Span, Mazes, Brief Visuospatial Memory Test-Revised, Fluency, Managing Emotions, Continuous Performance Test-Identical Pairs) scores were matched with age, gender and years of education by MCCB transfer software to obtain T scores in seven cognitive domains (34).

### Magnetic resonance imaging data acquisition

All participants underwent MRI on a 3.0 T Siemens Verio magnetic resonance imaging scanner (Erlangen, Germany). T1-weighted MPRAGE structural MRI scans took the following optimized acquisition parameters: repetition time (TR) = 2300 ms; echo time (TE) = 2.96 ms; inversion time (TI) = 900 ms; flip angle = 9°; voxel size = 1.0 mm × 1.0 mm × 1.0 mm; matrix size = 256 × 240 × 192; slice thickness = 1.00 mm; field of view (FOV) = 256 mm. During the scan, subjects should keep awake, eyes closed, head fixed, supine position quietly, and not perform specific cognitive tasks. Wear the earplugs to avoid scanner noise and reduce head motion.

### Cortical thickness and local gyrification index measurement

The T1-weighted images were processed using the FreeSurfer, an open neuroimaging toolkit, to automatically acquire measurement of cortical thickness and Local Gyrification Index (LGI) in each hemisphere (35). Cortical thickness and LGI calculations for each subject were run in the same version (5.3). The specific flow included: removal of non-brain tissue, bias field correction, tissue segmentation, cortical surface model reconstruction, rigorous data quality control. And LGI was calculated at an additional FreeSurfer processing stream (36). More details can be found on the FreeSurfer Wiki.^1^ Sixty-eight cortical regions (34 cortical regions in each hemisphere) were extracted as regions of interest for analysis in accordance with the Desikan–Killiany Atlas (37), and finally cortical thickness and LGI values were obtained.

### Statistical analyses

The demographic and clinical characteristics were compared between groups using Variance model (ANOVA), Student’s *t*-tests and Chi-square tests (SPSS version 25.0). The Least-Significant Difference (LSD) correction was used for multiple comparisons. When *P*-value was less than 0.05, the difference was statistically significant. One-way ANOVA analysis was used to explore differences of all cognitive domains and each hemisphere cortical thickness and LGI among AVH, non-AVH and HC groups. Partial Pearson’s correlation was used to investigate associations between HAHRS scores, cortical thickness or LGI, cognitive function in AVH group in order to explore the neural mechanisms and cognitive associations. Age, gender, years of education were taken as covariates.

## Results

### Demographic and clinical characteristics

Demographic and clinical characteristics of the study participants among AVH (*N* = 78), non-AVH (*N* = 74), and HC (*N* = 76) groups are presented in Table 1. The results indicated no significant differences in age, gender, years of education and handedness among three groups (All *p* > 0.05). However, both AVH and non-AVH groups showed significantly lower IQ compared to HC groups (LSD corrected *p* < 0.001). There were also no significant differences in education level, smoking, duration of untreated psychosis and family history between AVH and non-AVH groups (All *p* > 0.05). For cognitive functions, we found that schizophrenia patients with AVH or without AVH had notably poorer cognitive functions in all cognitive domains of MCCB than HC group (Table 2). Furthermore, AVH patients had more severe cognitive impairment in visual learning and verbal learning compared to non-AVH group (LSD corrected *p* < 0.05). The results of Student’s *t*-tests indicated no differences in PANSS subscales include positive, negative and general symptom, but the difference in PANSS-total score was statistically significant (*p* < 0.001).

**TABLE 1:** Comparisons of demographic and clinical characteristics in AVH, non-AVH, and HC groups.

	AVH (*N* = 78) Mean ± SD	Non-AVH (*N* = 74) Mean ± SD	HC (*N* = 76) Mean ± SD	*p/χ^2^*	AVH VS. HC *p*	Non-AVH VS. HC *p*	AVH VS. non-AVH *p*
Age (years)	24.83 ± 7.31	24.39 ± 7.58	25.24 ± 6.59	0.771	0.727	0.471	0.705
Gender (M/F)	49/29	47/27	46/30	0.924	–	–	–
Handedness (R/L)	78/0	74/0	76/0	–	–	–	–
Years of education	12.99 ± 0.31	13.50 ± 2.68	14.04 ± 2.67	0.054	0.016	0.220	0.240
Smoke (yes/no)	8/70	5/69	NA		–	–	0.441
DUP (months)	10.77 ± 7.56	11.43 ± 7.83	NA		–	–	0.596
Family history (yes/no)	6/72	10/64	NA		–	–	0.242
PANSS							
Positive	24.60 ± 3.10	22.72 ± 19.71	NA		–	–	0.418
Negative	20.26 ± 3.79	19.42 ± 4.08	NA		–	–	0.192
General	45.81 ± 4.35	44.57 ± 3.39	NA		–		0.053
Total	90.46 ± 8.93	84.42 ± 7.42	NA		–		0.000*
HAHRS	21.03 ± 3.09	0	NA		–		

AVH, schizophrenia patients with auditory verbal hallucination; non-AVH, schizophrenia patients without auditory verbal hallucination; PANSS, the Positive and Negative Syndrome Scale; HC, healthy control; DUP, duration of untreated psychosis; HAHRS, Hoffman Auditory Hallucination Rating Scale. All *p* are LSD corrected (*p < 0.001).

**TABLE 2:** Comparisons of IQ and cognitive function in three groups.

	AVH *N* = 78	Non-AVH *N* = 74	HC *N* = 76	*p*	AVH vs. HC *p*	Non-AVH vs. HC *p*	AVH vs. Non-AVH *p*
**WAIS(IQ)**	103.42 ± 16.90	107.61 ± 12.67	115.93 ± 9.02	0.000*	0.000*	0.000*	0.049
**MCCB**							
**Speed of processing**	37.23 ± 12.96	38.64 ± 10.34	51.42 ± 8.94	0.000*	0.000*	0.000*	0.428
**Attention/vigilance**	37.76 ± 10.00	38.16 ± 11.84	48.24 ± 7.57	0.000*	0.000*	0.000*	0.802
**Working memory**	35.21 ± 9.45	35.85 ± 10.89	45.24 ± 7.72	0.000*	0.000*	0.000*	0.670
**Visual learning**	39.47 ± 12.03	43.46 ± 11.96	51.51 ± 7.53	0.000*	0.000*	0.000*	0.023
**Verbal learning**	35.38 ± 11.24	38.93 ± 11.04	47.16 ± 9.54	0.000*	0.000*	0.000*	0.041
**Reasoning/problem solving**	46.12 ± 11.01	45.28 ± 11.30	53.58 ± 8.03	0.000*	0.000*	0.000*	0.617
**Social cognition**	32.26 ± 10.47	34.58 ± 12.07	39.59 ± 9.96	0.000*	0.000*	0.000*	0.188
**Total**	29.90 ± 12.93	32.65 ± 12.44	46.95 ± 8.38	0.000*	0.000*	0.000*	0.140

IQ, intelligence quotient; MCCB, MATRICS consensus cognitive battery. All *p* are LSD corrected (*p < 0.001).

### Comparisons of cortical thickness

**FIGURE 1:**
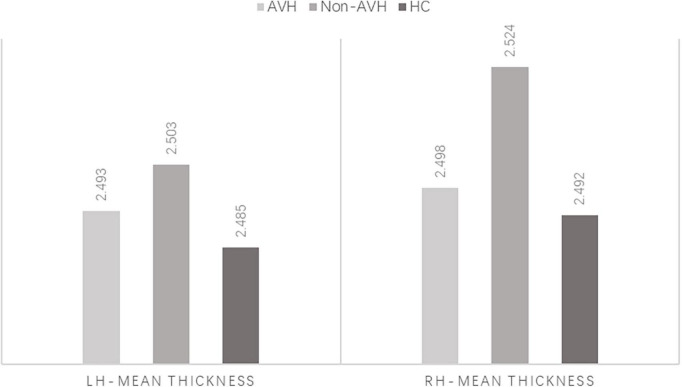
Mean cortical thickness of the left and right hemisphere. lh, left hemisphere; rh, right hemisphere.

**FIGURE 2:**
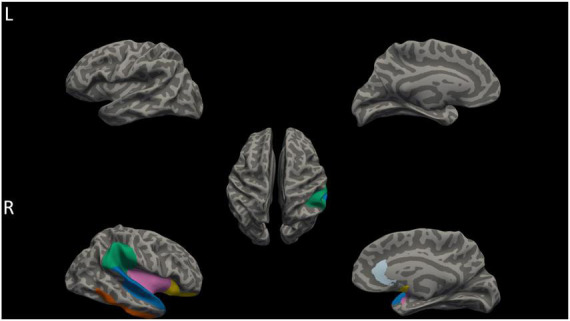
Differences in cortical thickness among AVH, non-AVH, and HC groups. The right hemisphere of STG, ITG, SMG, lateral OFC, rACC, and INS (labeled in blue, orange, green, yellow, sky blue, and pink) showed significant differences in cortical thickness among three groups (*p* < 0.05, corrected for multiple comparisons with LSD). L, left; R, Right.

Compared to HC group, mean cortical thickness of patients group was thicker (HC < AVH < non-AVH), and three groups significantly differed on the right hemisphere (rh)-mean cortical thickness (LSD corrected *p* < 0.05) (Figure 1). Among three groups, our results demonstrated that the inferior temporal gyrus (ITG), superior temporal gyrus (STG), lateral orbito frontal cortex (OFC), rostral anterior cingulate cortex (rACC), supramarginal gyrus (SMG), and insula (INS) in the right hemisphere were found to have significant differences (all LSD corrected *p* < 0.05) (Figure 2). Thinner cortex in six regions was explored between schizophrenia patients with AVH than non-AVH. In addition, the AVH group exhibited thinner cortical thickness in the right ITG, right STG, and right SMG compared with HC group (Table 3).

**TABLE 3:** Comparisons of cortical thickness among three groups.

Cortical thickness				F	*p*	AVH vs. HC	Non-AVH vs. HC	AVH vs. non-AVH
	AVH	Non-AVH	HC			*p*	*p*	*p*
rh-ITG	2.82 ± 0.11	2.89 ± 0.13	2.83 ± 0.10	3.701	0.026	0.508	0.052	0.009
rh-STG	2.86 ± 0.13	2.91 ± 0.14	2.86 ± 0.13	3.299	0.039	0.907	0.023	0.030
rh-lateral OFC	2.67 ± 0.13	2.72 ± 0.13	2.66 ± 0.11	4.538	0.012	0.445	0.004	0.031
rh-rACC	2.72 ± 0.16	2.79 ± 0.20	2.70 ± 0.17	5.391	0.005	0.670	0.003	0.009
rh-SMG	2.52 ± 0.12	2.57 ± 0.11	2.54 ± 0.12	3.785	0.024	0.184	0.158	0.006
rh-INS	3.01 ± 0.13	3.06 ± 0.13	2.98 ± 0.12	7.736	0.001	0.199	0.000*	0.010
rh-MT	2.50 ± 0.08	2.52 ± 0.09	2.49 ± 0.07	3.295	0.039	0.659	0.017	0.048

rh, right hemisphere; ITG, the inferior temporal gyrus; STG, superior temporal gyrus; OFC, orbito frontal cortex; rACC, rostral anterior cingulate cortex; SMG, supramarginal gyrus; INS, insula; MT, Mean thickness. All *p* are LSD corrected (*p < 0.001).

### Comparisons of local gyrification index

**FIGURE 3:**
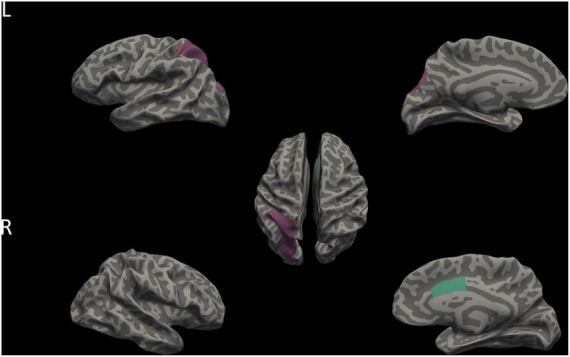
Differences in LGI among AVH, non-AVH, and HC groups. Purple and aqua green indicates two brain regions with significant differences in LGI among three groups (*p* < 0.05, corrected for multiple comparisons with LSD). L, left; R, Right.

We calculated LGI within the 34 cortical regions in each hemisphere. Our results showed LGI in the left hemisphere of superior parietal gyrus (SPG) and the right hemisphere of caudal anterior cingulate cortex (CAC) was significantly different among AVH, non-AVH, and HC groups (both LSD corrected *p* < 0.05; see Table 4). Furthermore, we found that higher LGI on the left SPG and the right CAC in AVH groups (Figure 3).

**TABLE 4:** Comparisons of LGI among three groups.

	AVH	LGI non-AVH	HC	F	*p*	AVH VS. HC *p*	Non-AVH VS. HC *p*	AVH VS. non-AVH *p*
lh-SPG	3.20 ± 0.15	3.14 ± 0.15	3.19 ± 0.13	3.790	0.024	0.507	0.049	0.009
rh-CAC	2.12 ± 0.12	2.07 ± 0.11	2.07 ± 0.11	3. 449	0.033	0.025	0.964	0.023

lh, left hemisphere; rh, right hemisphere; LGI, Local Gyrification Index; SPG, superior parietal gyrus; CAC, caudal anterior cingulate. All *p* are LSD corrected.

### Associations between the severity of AVH and cognitive function, cortical thickness, or local gyrification index

**FIGURE 4:**
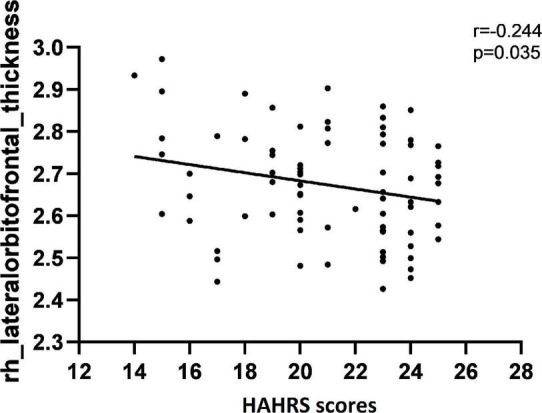
The cortical thickness of rh-lateral OFC was negatively correlated with the severity of auditory verbal hallucination, as assessed by HAHRS scores.

**FIGURE 5:**
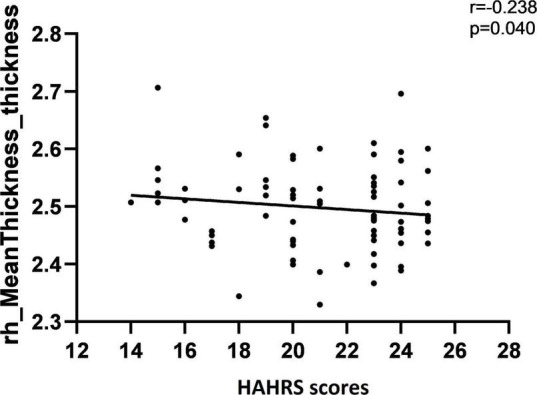
The mean cortical thickness in the right hemisphere was negatively correlated with the severity of auditory verbal hallucination, as assessed by HAHRS scores.

The correlations of auditory verbal hallucination, cognitive function, and cortical thickness or LGI were reported with age, gender, and years of education as covariates to assess the relationship between these variables. The subsequent correlation analyses conducted in AVH group only. In terms of the associations between auditory verbal hallucination and cortical thickness or LGI values, the cortical thickness of rh-lateral OFC was negatively correlated with the HAHRS scores (*r* = −0.244, *p* = 0.035) (Figure 4). The HAHRS scores also showed negative correlations with the rh-mean thickness (*r* = −0.238, *p* = 0.040) (Figure 5), and no significant associations were found with the LGI values.

Regarding the relationships between auditory verbal hallucination and cognitive function, there was not a significant trend toward for the correlation between AVH severity and cognition (All *p* > 0.05).

Additionally, correlation analyses between cognitive function and cortical thickness or LGI values revealed no significant associations. Beyond that, we also conducted in HC and non-AVH groups, the results still showed no significant correlations.

## Discussion

Our present study aimed to reveal brain structural abnormalities and cognitive function differences between first-episode drug-naïve schizophrenia patients with and without auditory verbal hallucination and investigate the mutual relationships among brain regions with altered cortical thickness or local gyrification index, cognitive deficit, the severity of AVH in schizophrenia patients. The main findings emerged: (1) both patients with and without AVH had extensive cognitive deficits compared to HC group. Interestingly, the AVH patients had even more prominent cognitive impairment than non-AVH group, especially in visual learning and verbal learning domains. (2) There were significant differences in brain structure indexes among three groups, mainly manifested as follows: AVH group showed cortical thinning in the right hemisphere of STG, ITG, SMG, lateral OFC, rACC, and INS. Additionally, AVH patients showed increased LGI in the left SPG and the right CAC compared to non-AVH patients. (3) The cortical thickness on the right lateral OFC was negatively correlated with the severity of AVH in schizophrenia patients with AVH.

### Cognitive dysfunction in schizophrenia patients with AVH

Consistent with the results of most previous studies, our results also demonstrated that first-episode and drug-naïve schizophrenia patients had notably and extensive poorer cognitive function compared to control subjects. Moreover, we further found that AVH patients had more severe cognitive impairment in visual learning and verbal learning compared to non-AVH patients. This is partially consistent with previous studies which suggested that compared to never voice-hearers, current and past voice-hearers are more likely to encounter difficulties in integrating new information on the visual learning domain, but not significant more impaired in verbal learning (38, 39). However, other studies have exhibited that poorer performance on verbal working memory was identified as the independently predictor of the severity of AVH in first-episode schizophrenia patients (7). Hopkins Verbal Learning Test (HVLT) and Brief Visuospatial Memory Test (BVMT) from MCCB were used separately to assess verbal learning and visual learning at present study. Both tasks are purposed to detect subtle learning deficits, perhaps fluctuating in line with AVH or other clinical symptom severity and even influenced by inter individual differences. A possible interpretation for the two cognitive deficits of verbal learning and visual learning could be more state-based, inversely, other cognitive domains could be trait-based. The phonological loop required for verbal learning was disturbed by AVH, which results in neuronal resources of the auditory cortex being under occupation by internally produced phonological representations (7, 40, 41). Based on the previous studies and our results, AVH may exacerbate partial cognitive deficits in schizophrenia, but more studies are needed to clarify the relationship between them in the future.

### Cortical thickness abnormalities in schizophrenia patients with AVH

Ample evidence supports that altered cortical thickness in the frontal, temporal, parietal, cingulate gyrus, and INS in schizophrenia patients compared to the control subjects, and was related with their clinical symptoms (14, 17, 19). In the present study, we found that patients with schizophrenia had increased cortical thickness in the right hemisphere of STG, ITG, SMG, lateral OFC, rACC, and INS compared to HC based on the symptoms of AVH. Significantly, patients without AVH showed the thickest cortex under compares, followed by patients with AVH. A reliable explanation is compensatory increases in cortical thickness at the initial stages of schizophrenia, and destructive changes in cortex structure in schizophrenia with AVH (42). This finding is concordant with Guo et al. who reported a compensatory rebuilding process result in the cortical thickness thickening in different periods of schizophrenia (43).

Moreover, Cortical morphological abnormal areas associated with AVH have been implicated across prior neuroimaging studies in schizophrenia, including gray matter loss in the anterior/posterior cingulate, frontal, temporal, and insular cortex (44, 45), which are approximately consistent with our results. Data from a voxel-based morphometry study demonstrated that schizophrenia accompanied with AVH showed six cortical clusters reductions located in the bilateral STG, left SMG, left posterior cingulate cortex and left postcentral gyrus (44). There is growing AVH evidence which reported a negative correlation between the volume of the STG and AVH proneness (13, 46–48). Hence, we anticipated that these abnormal brain regions could be neural targets for clinical interventions in AVH.

Early evidence demonstrates that cortex thinning in schizophrenia patients over time throughout the disease courses (49), and antipsychotic drugs could relieve clinical symptoms by regulating the regional cerebral volume (50). In the present study, we recruited first-episode drug-naïve schizophrenia patients so as to reduce the influence of confounding factor on cortical thickness to explore the relationship between the severity of AVH and cortical thickness. Our results found a negative correlation between the severity of AVH and variations of mean cortical thickness in the right hemisphere, especially in the right lateral OFC. To the best of our knowledge, although substantial neuroimage studies conducted in schizophrenia, only a few for auditory hallucinations in those patients and also with inconsistent conclusions. A recent study indicated that schizophrenia patients with AVH exhibited greater reductions in gray matter volumes in the frontal, temporal, cingulate and insular areas, but they were not correlated with the severity of the AVH (51). Another study found reductions in cortical thickness in the left hemisphere, not the right hemisphere in schizophrenia patient with AVH (46). Due to limited relevant studies, further researches are warrant to replicate our findings.

### Local gyrification index abnormalities in schizophrenia patients with AVH

To study the diversity of the phenomenological characteristics of AVH based on the prior neuroimaging studies, we also investigated the relationship between LGI, a neurodevelopmental marker of brain structure, and AVH in schizophrenia patients. Our results showed that schizophrenia patients with AVH had increased LGI in the left SPG and the right CAC compared to non-AVH patients and healthy controls, which was not in line with other previous studies. An early study demonstrated hypogyria in the bilateral posterior cingulate and bilateral CAC in schizophrenia patients with AVH compared to non-AVH patients (26). A recent study also found abnormal LGI common to AVH and non-AVH groups in ACC area, in addition, increased LGI in the precuneus and SPG were found in schizophrenia patients with persistent AVH, which is partially the same as our result (52). It has been suggested that increasing local gyrification with decreasing cortical thickness, whereas their correlation in AVH is still unclear (23, 53). These conflicting results could be due to different assessment tools and high heterogeneity, including different age at onset, duration of illness, years of education and frequency of psychotic episode. Future studies with larger sample size and more confounding factors controlled should be conducted to supplement the evidence in this regard.

## Limitation

The current study has several limitations. First, given the cross-sectional nature of this research, we cannot draw a causal conclusion between AVH and cognitive performance or cortex structural changes on our results. Hence, future studies combined with longitudinal design are warranted to dynamically observe the developmental context of brain structural and cognitive functional alterations in patients with AVH. Second, we divided schizophrenia into with and without AVH, resulting in a small size of sample of subgroups. Finally, based on the analysis method of multimodal image data to explore AVH in schizophrenia could further clarify the pathogenetic mechanism of AVH.

## Conclusion

In summary, our results indicated that schizophrenia patients with AVH had more severe cognitive impairment, especially on verbal learning and visual learning domains, compared to non-AVH patients, and patients with AVH also had specific cortical and LGI features in brain compared to non-AVH patients. Furthermore, the cortical thickness in the right lateral OFC was negatively correlated with the AVH severity in patients with AVH. Taken together, our preliminary findings provided evidence suggest that cortical structural abnormalities may be associated with the cognitive dysfunction and AVH in schizophrenia patients. Further studies are warrant to verify our findings and explore the potential mechanism underlying this result.

## Data availability statement

The raw data supporting the conclusions of this article will be made available by the authors, without undue reservation.

## Ethics statement

The studies involving human participants were reviewed and approved by the Medical Research Ethics Committee of the Affiliated Brain Hospital of Nanjing Medical University. Written informed consent to participate in this study was provided by the participants’ legal guardian/next of kin. Written informed consent was obtained from the individual(s), and minor(s)’ legal guardian/next of kin, for the publication of any potentially identifiable images or data included in this article.

## Author contributions

XS, RZ, and SX designed the study. XS, FJ, XF, WY, and RZ recruited the participants and completed the data collection. XS, FJ, and XF analyzed the data and wrote the manuscript. RZ and SX revised the manuscript. All authors reviewed the manuscript and approved the final manuscript.

## Footnotes

1
http://surfer.nmr.mgh.harvard.edu/
